# What type of tremor did the medieval ‘Tremulous Hand of Worcester’ have?

**DOI:** 10.1093/brain/awv232

**Published:** 2015-08-31

**Authors:** Deborah E. Thorpe, Jane E. Alty

**Affiliations:** 1 1 Centre for Chronic Diseases and Disorders (C2D2)/ Electronics Department, University of York, UK; 2 2 Hull York Medical School, University of York, UK; 3 3 Neurology Department, Leeds Teaching Hospitals NHS Trust, UK

## Abstract

The thirteenth-century medieval scribe, the ‘Tremulous Hand of Worcester’ is known for the tremor visible in his script. Thorpe & Alty combine historical analysis with the first neurological study of the scribe’s handwriting. After considering various differential diagnoses, they conclude that the balance of evidence favours essential tremor.

## Graphical abstract

**Figure:**
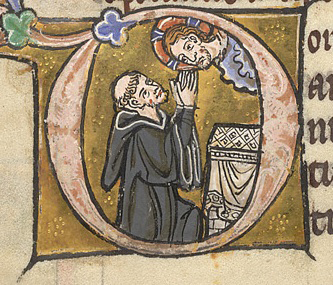
The thirteenth-century medieval scribe, the ‘Tremulous Hand of Worcester’ is known for the tremor visible in his script. Thorpe & Alty combine historical analysis with the first neurological study of the scribe’s handwriting. After considering various differential diagnoses, they conclude that the balance of evidence favours essential tremor.

## Introduction

Scholars have recognized for some time that a prolific 13th century scribe had a tremor. He has become known as ‘the Tremulous Hand of Worcester’, or simply ‘the Tremulous Hand’, ‘hand’ being a metonym for ‘scribe’. He is important as the only widely-known medieval writer with a tremor, and for his unusual interest in translating documents written centuries earlier. This is the first time his writing has been investigated from a joint neurological and historical perspective.

Certain or possible evidence of the writing of this man—likely a monk at Worcester Cathedral Priory—appears in at least 20 books (Franzen, 1991 p. 1). As he never wrote about his tremor, or dated his work, the only sources of information for this study are the handwriting itself and limited clues in its subject matter.

The central question is: ‘what type of tremor did he have?’. We discuss evidence for essential tremor as the diagnosis by tracing the tremor through a series of handwriting samples, charting progression in tremor severity from ‘fine’ to ‘fine–moderate’ and as a correlate, present handwriting from a modern-day individual with essential tremor using a calligraphy pen. We scrutinize literary scholar Christine Franzen’s seminal monograph, reveal new information she has shared with us in personal correspondence, and offer the first analysis of essential tremor in a medieval context. To our knowledge, this is the first time medieval handwriting has been analysed by a neurologist with a specialist interest in movement disorders. Finally, we examine the lifestyle of a scribe in relation to the symptoms of essential tremor, making special consideration of alcohol consumption.

## The annotations

**Figure 1:**
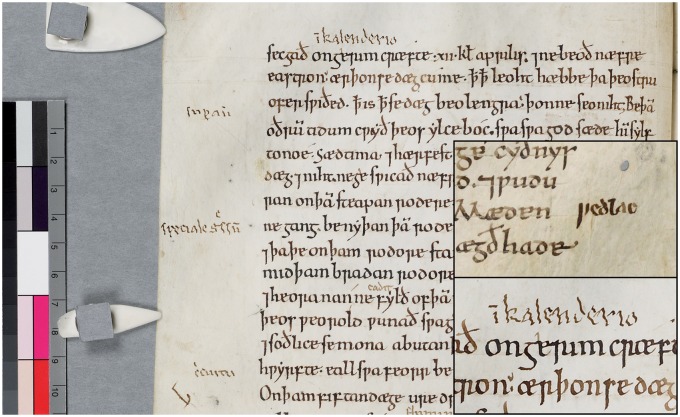
**Image displaying later annotations in Latin by the Tremulous Hand of Worcester, visible in the margins and interlinear to the main text.** Cambridge, Corpus Christi College Manuscript 178, p. 22, lines 1–15. *Inset* (*top*): Detail of annotation in Middle English, designated as early by Franzen. Cambridge, Corpus Christi College, Manuscript 198. Folio 71^r^. *Inset* (*bottom*): Detail of annotation in Latin, designated as late by Franzen. By permission of the Master and Fellows of Corpus Christi College, Cambridge. This image/content is not covered by the terms of the Creative Commons licence of this publication. For permission to reuse, please contact the rights holder.

Old English was the earliest form of English, spanning approximately the 6th to 12th centuries, which had been superseded by Middle English by this scribe’s lifetime. Thus, he translated older books into either Middle English or French, switching to Latin later in his life (Franzen, 1991: pp. 2–4, 27–8, 101–2; Fig. 1). He also numbered chapters and altered previously incorrect numbers (Franzen, 1991: p. 67).

The scribe’s annotations provide indications of his interests; he made Latin *nota* marks in the margins, a contraction of *nota bene* or ‘note well’ (Franzen, 1991: p. 29). Among the subjects he annotated were health issues, as is seen in one collection of Old English herbal remedies, in particular, urinary and eye ailments (Franzen, 1991: pp. 67–8).

Franzen provided a letter from medical historian Margaret Pelling pointing out that the incidence of bladder stones was higher in the medieval period, particularly in individuals with sedentary occupations (Franzen, 1991: p. 199). This is relevant, as presumably this scribe spent many hours stationary. Alternatively, a unifying diagnosis of tremor with bladder problems might suggest Parkinson’s disease. Franzen suggested that the increasing size of his handwriting in its later stages could corroborate failing eyesight (Franzen, 1991: pp. 2, 68). Visual impairment would have worried the scribe, for whom reading and writing were important (Pelling, personal communication; Franzen, 1991: p. 199) and Parkinson’s disease, cerebellar tremors and thyrotoxicosis, comprising tremor and coexistent visual symptoms, should also be considered.

The scribe also marked remedies for *hramma* (cramp or spasms) (Franzen, 1991: pp. 68, n.66), which invites further enquiry, since tremor associated with cramp might suggest a dystonic or Parkinsonian tremor or writer’s cramp. However, he drew attention to other conditions too such as warts, nose-bleeds, and earache (Franzen, 1991: p. 68), leaving it unclear if cramp was especially worrying for him, or just one of many concerning ailments.

## Chronology

**Figure 2:**
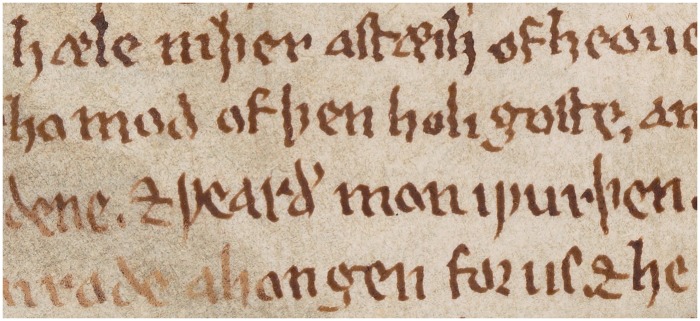
**A reduction in the severity of the scribe's tremor at lines 3 and 4, apparently due to rest (Franzen, 1991, p. 57), or possibly alcohol-related.** Detail of Oxford, Bodleian Library, Manuscript Junius 121, Folio vi^r^. By permission of The Bodleian Libraries, University of Oxford. This image/content is not covered by the terms of the Creative Commons licence of this publication. For permission to reuse, please contact the rights holder.

Franzen originally discerned seven chronological ‘layers’ of writing, but acknowledged variation within each layer (1991: pp. 5, 15–9, 27–8), perhaps because the scribe wrote under different degrees of fatigue. Indeed, there is one instance where he evidently took a break after writing nine lines, returning to work at the beginning of the 10th with a steadier script (Fig. 2; Franzen, 1991: p. 57).

We can reveal that Franzen has re-evaluated her original chronology, stating in personal correspondence that distinguishing seven layers was perhaps ‘over zealous’ (Franzen, personal communication, October 2014). She now notes a shift from early stage annotations in Middle English—upright and lacking signs of an obvious tremor—to later stage annotations in Latin, with their marked leftward lean and pronounced tremor (Fig. 1).

It is in the earliest layers that we see most errors and the scribe often returned later to correct inaccurate glosses (Franzen, 1991: pp. 15–9, 98, 101). His spelling ability was consistently good, so his early errors appear to stem from inexperience, unfamiliarity with certain words, or difficulties in translating archaic language rather than cognitive difficulties (Franzen, 1991: pp. 173–82).

## Previous work on the tremor

Franzen’s discussion of the tremor comprises just one and a half pages, and no other scholars have discussed it in detail. Franzen eliminated Parkinson’s disease, citing general practitioner and medical historian Dr Irvine Louden’s statement that it would produce ‘erratic and uneven writing’ rather than the ‘fine, regular tremor’ seen here (1991: p. 198) and there was no micrographia. This warrants clarification, as Parkinson’s disease tremor frequency is typically regular and micrographia not universal (Wagle Shukla *et al.*, 2012).

Louden also advised: ‘the progressive lean to the left, the “splayed” look, the failure to join up strokes, and the exaggerated size of the writing are all characteristics of a congenital tremor in the later stages’ (Franzen, 1991: p. 198). ‘Congenital tremor’ is ambiguous, as dystonic, essential, and cerebellar tremors, amongst others, could all be considered congenital. Therefore, the ‘evaluating the handwriting’ section below augments these assertions with an exhaustive study of the features that indicate a more precise tremor classification.

## Evaluating the handwriting

Inspection of the Tremulous Hand’s writing provides useful diagnostic information: evaluating the graphic features of letters affected by tremor and how they change over time. We acknowledge that we do not know whether he had unilateral or bilateral upper limb tremor, or whether other body parts were tremulous. Similarly, the tremor may only have affected writing, so primary writing tremor and writer’s cramp are considered in the differential, especially as scribes would be at particular risk of task-specific tremor/dystonia. We also do not know his age; Franzen argued that the tremor was not attributable to old age (1991: pp. 2, 198–9), and has suggested he was not elderly in personal correspondence, pointing to the lack of deterioration in his mental abilities. However, people who reached 40 in this period could expect to live for another 30 or 40 years (Shahar, 2005: p. 71). A lack of evidence for cognitive decline would not preclude the possibility that this scribe was elderly, thus we argue that he may already have reached an advanced age when he wrote the earlier samples. The absence of evidence of cognitive decline points away from Parkinson’s disease, or fragile X-associated tremor/ataxia syndrome.

The contour of letters demonstrates a regular amplitude tremor—individual letters within words have the same degree of lateral deviation. The amplitude is best described as ‘fine’ to ‘fine–moderate’ as lateral deviations are small relative to the letter size (Figs 1 and 2). The tremor frequency appears regular too, with the number of lateral crossings about the midline on downward strokes consistent between letters of roughly the same size within a word, or consecutive words. This regularity of tremor amplitude and frequency makes dystonic tremor and writer’s cramp less likely. Figures 1 and 2 demonstrate a single tremor axis—the deviation of ink is in a consistent direction—and this tends to discriminate essential from dystonic tremor (Michalec *et al.*, 2014). The axis, from approximately 8 pm to 2 pm, with tremor most prominent in the vertical sections of letters, suggests a distal (e.g. essential tremor, dystonic tremor, Parkinson’s disease, primary writing tremor), rather than proximal (e.g. Holmes and cerebellar) tremor, in a right-handed person (Wang *et al.*, 2005). The axis never switches over to an opposite direction (i.e. from 11 pm to 5 pm), as might be seen in writer’s cramp when the left hand is used.

Individual letters are regular in size and shape despite the tremor with no evidence of ataxia, as might be seen with a cerebellar tremor, Holmes tremor, or fragile X-associated tremor/ataxia syndrome. Furthermore the nib pressure appears steady, rather than forceful, as often occurs with dystonic posturing.

Interestingly, the tremor amplitude progresses from ‘fine’ to ‘fine–moderate’ over time (Fig. 1 and inset). This is consistent with Elble *et al.*’s (2000) study that demonstrated essential tremor frequency declined by ∼0.08 Hz per year with concurrent augmentation in amplitude.

It is difficult to measure tremor frequency from a historical document as we cannot know the quill speed during writing. Frequency, nonetheless, can be estimated by counting the number of crossings about the midline for letters with long downstrokes of similar height such as ‘l, h, k’ and ‘p’. Assuming each downstroke took less than half a second, one can surmise that the frequency was at least 6–8 Hz because there are typically three to four complete oscillations about the midline i.e. one movement to the right, one to the left, and back to the midline. The frequencies of typical essential, dystonic, and Parkinson’s disease tremors are 8–10 Hz,≤7 Hz, and 4–6 Hz, respectively, so an estimate of 6–8 Hz sits ambiguously among these. To complicate matters, several ‘atypical’ action tremors, with higher frequencies, have also been described in Parkinson’s disease. Nevertheless, although overlapping tremor frequencies are acknowledged; Burne *et al.* (2002) demonstrated using EMG that frequencies >6 Hz tend to be essential tremor rather than Parkinson’s disease. Certainly, 6–8 Hz would not be consistent with Holmes and cerebellar tremor frequencies.

The absence of micrographia or decrementing amplitude, even in later entries, and samples comprising multiple lines (Fig. 2), is noteworthy. Although not all patients with Parkinson’s disease exhibit these handwriting features (Wagle Shukla *et al.*, 2012), their absence would suggest an alternative diagnosis. However, it could be argued that decrementing amplitude would not develop when only single words or a few words are written, rather than long full sentences.

Another intriguing feature is the apparent rapid improvement in tremor severity part way through one sample of the scribe’s writing. Franzen believes he took a break and his writing improved considerably upon his return (Fig. 2; Franzen, 1991: p. 57). Tremor improvement after rest, or relaxation, has been reported in Parkinson’s disease, dystonic tremor, essential tremor, writer’s cramp, and primary writing tremor. Perhaps the scribe consumed alcohol during the break; it was typical for medieval people to hydrate themselves with weak alcohol throughout the day (Unger, 2004: p. 3). The striking improvement may be the characteristic alcohol-response of essential tremor, or the lesser known alcohol-response of dystonic or primary writing tremor (Haubenberger *et al.*, 2011).

**Figure 3:**
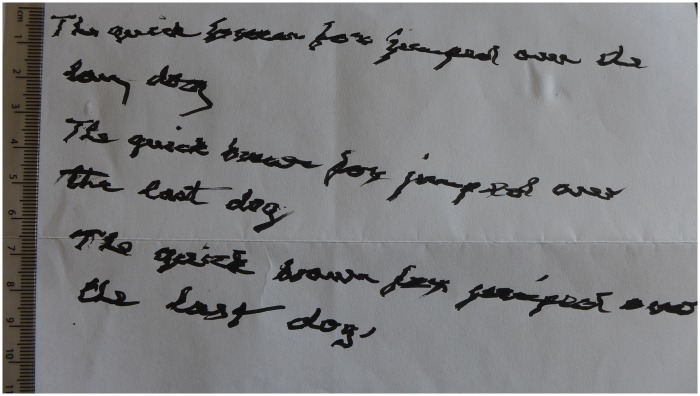
**Example of modern-day handwriting made using a pen with a calligraphy nib from a man with a 15-year history of essential tremor**.

As a correlate, a modern-day sample of handwriting was collected from a 78-year-old right-handed man with confirmed essential tremor using a calligraphy pen (Fig. 3). There are several commonalities between the modern and medieval samples: unidirectional 8 pm to 2 pm axis, consistent degree of tremor amplitude across several lines, and similar tremor frequencies—i.e. three complete oscillations in the vertical strokes of equivalent sized letters such as the ‘b’ in ‘brown’ (Fig. 3) and the ‘l’ and ‘k’ in the medieval text (Fig. 1). In Fig. 3 the tremor amplitude is coarser than the fine tremor seen in the earlier medieval scripts but similar to the later annotations (Fig. 1), perhaps because the modern-day writer has had essential tremor for 15 years.

## Conclusions

We conclude that essential tremor, dystonic tremor, and primary writing tremor are the main differential diagnoses for the Tremulous Hand’s tremor. We doubt that a scribe would be so prolific if afflicted with writer’s cramp and there is no evidence of dystonic posturing. Table 1 summarizes the evidence relating to the remaining three diagnoses. The weight of the evidence balances towards essential tremor. This is based on evidence of a fine amplitude, regular frequency tremor, of at least 6–8 Hz, with a unidirectional axis, that was present during action and exhibited rapid fluctuations in severity. The lack of evidence of cognitive decline, amplitude decrement, micrographia, ataxia, or increased nib pressure, is consistent with this diagnosis. Furthermore, essential tremor is more prevalent than the other diagnoses. Although the scribe drew attention to bladder and visual symptoms, and these are not known associations of essential tremor, their significance is speculative. Even if he was afflicted by these problems, they may have been coexistent pathologies rather than part of a unifying tremor syndrome. The authors welcome the readers’ responses to the evidence presented, through the ‘Letters to the Editor’ section of the journal.

**Table 1:** Comparing the evidence from the Tremulous Hands annotations for each of the three differential diagnoses

Handwriting feature	Evidence from the Tremulous Hand’s entries	Typical features		
		Essential Tremor	Dystonic Tremor	Primary Writing Tremor
Tremor amplitude	Fine (early) and fine–moderate	Fine or fine–moderate	Moderate	Large
Tremor frequency (Hz)	At least 6–8	8–12	<7	5–7
Change over time	Tremor amplitude increases	Yes	Possibly	No
	Size of letters increases	No	Yes	Sometimes
Alcohol response	Possible^a^	Yes	Sometimes	Sometimes
Rest response	Possible^a^	Yes	Yes	Yes
Eye symptoms	Speculative	No	Rarely	No
Bladder symptoms	Speculative	No	No	No
Excessive handwriting as a risk factor?	Yes (scribe)	No	Yes	Yes
Cognitive impairment	None	Possibly	Rarely	No
Dystonic posturing	None	No	Yes	Sometimes
Micrographia	None	No	No	No

^a^Evidence of sudden improvement in tremor severity.
